# Predicting the characteristics of the aetiological agent for Kawasaki disease from other paediatric infectious diseases in Japan

**DOI:** 10.1017/S0950268815001223

**Published:** 2015-07-23

**Authors:** Y. NAGAO, C. URABE, H. NAKAMURA, N. HATANO

**Affiliations:** 1 Japan Community Health Care Organization, Osaka Hospital, Fukushima, Osaka, Japan; 2 Institute of Industrial Science, the University of Tokyo, Komaba, Meguro, Tokyo, Japan; 3 National Institute for Fusion Science, Oroshi-cho, Toki, Gifu, Japan

**Keywords:** Emerging infections, epidemiology, infectious disease epidemiology, Kawasaki disease, mathematical modelling

## Abstract

Although Kawasaki disease (KD), which was first reported in the 1960s, is assumed to be infectious, its aetiological agent(s) remains unknown. We compared the geographical distribution of the force of infection and the super-annual periodicity of KD and seven other paediatric infectious diseases in Japan. The geographical distribution of the force of infection, which was estimated as the inverse of the mean patient age, was similar in KD and other paediatric viral infections. This similarity was due to the fact that the force of infection was determined largely by the total fertility rate. This finding suggests that KD shares a transmission route, i.e. sibling-to-sibling infection, with other paediatric infections. The super-annual periodicity, which is positively associated with the sum of an infectious disease's incubation period and infectious period, was much longer for KD and exanthema subitum than other paediatric infectious diseases. The virus for exanthema subitum is known to persist across the host's lifespan, which suggests that the aetiological agent for KD may also be capable of persistent infection. Taken together, these findings suggest that the aetiological agent for KD is transmitted through close contact and persists asymptomatically in most hosts.

## INTRODUCTION

Kawasaki disease (KD), which is the most common cause of paediatric vasculitis in developed countries, was first reported in Japan in the 1960s [1]. Following three large epidemics of KD, which occurred in 1979, 1982 and 1986, the incidence of KD has been steadily increasing in Japan [2]. Although the aetiology of KD has not been identified, KD is assumed to be infectious based on the presence of seasonality [3] and spatio-temporal clustering during epidemics [4]. In addition, KD shares many clinical manifestations (e.g. fever, conjunctivitis, rash, cervical adenopathy) with other viral infections [5]. The rarity of KD in infants aged <6 months indicates the presence of an effective passive immunity [6]. The recurrence of KD is rare [7], which suggests that KD may be caused by a limited number of – if not a single – aetiological agents. In recent years, a number of large-scale epidemiological studies have enhanced our understanding of the genetic susceptibility to KD [8–10]. By contrast, however, the aetiological agent(s) of KD has not been identified.

KD can cause a coronary artery lesion and a subsequent fatal myocardial infarction in 1–2% of patients [11, 12], unless treated promptly with high-dose intravenous immunoglobulin (IVIG) [13]; however, 40% of Japanese KD patients who are treated with IVIG therapy still develop transient coronary artery lesions [14]. IVIG therapy is not only expensive but also harbours the potential for several adverse effects. The identification of the aetiological agent for KD is therefore critical to the development of less expensive and more specific therapies. In the present study, we compared KD with other paediatric infectious diseases with known aetiologies in order to characterize the likely aetiology of KD. A comparison of the epidemiological profiles of multiple diseases can shed light on the determinants of transmission of the individual diseases [15]. Here, we focused on two epidemiological characteristics, the geographical distribution of the force of infection (FOI) and the periodicity, for KD and each of the seven other paediatric infections.

## METHODS

### KD data

We obtained the KD data from the Nationwide Surveillance of Kawasaki Disease (NSKD), which includes almost all of the reported cases of KD stratified by age across all 47 prefectures of Japan [16] since 1979 [17].

### Paediatric infectious disease data

The data for nine infectious diseases stratified by age have been reported weekly to the National Epidemiological Surveillance of Infectious Diseases (NESID) from approximately 3000 sentinel paediatric clinics since 1999 (e.g. 2978 clinics in 2000 and 3028 clinics in 2010) [18]. Our analysis excluded infectious gastroenteritis, which is caused by multiple heterogeneous pathogens, and mumps, for which the information about vaccine coverage cannot be obtained. Therefore, we analysed the data for seven paediatric infectious diseases (Table 1). Because the allocation of sentinel clinics is not precisely proportional to the population size, the absolute incidence of disease cannot be estimated from these data. Instead, we estimated the mean patient age from the patient age distribution (see Supplementary Fig. S1).

**Table 1.** Kawasaki disease and paediatric infectious diseases compared in the present study and their mean patient ages (years) in Japan measured between 2000 and 2010

Disease	Main aetiological agents	Vaccine	National crude mean age	National adjusted mean age	Range of prefectural crude mean age	Range of prefectural adjusted mean age
Kawasaki disease	Unknown	Not available	2·6	2·4	2·1–2·7	2·1–2·6
Exanthema subitum*	Human herpes virus 6 & 7	Not available	0·91	0·91	0·78–1·1	0·78–1·1
Herpangina	Enteroviuses	Not available	3·4	3·4	2·3–4·3	2·4–4·2
Hand, foot and mouth disease	Enteroviuses	Not available	3·7	3·6	2·5–4·1	2·5–4·1
Chickenpox	Varicella-zoster virus	Voluntary	3·7	3·7	3·1–4·3	3·0–4·3
Pharyngoconjunctival fever	Adenoviruses	Not available	4·6	4·5	3·5–5·7	3·4–5·7
Erythema infectiosum	Human parvovirus B19	Not available	6·1	6·1	5·5–6·7	5·4–6·7
Group A streptococcus	*Streptococcus pyogenes*	Not available	7·1	7·1	6·1–8·4	6·1–8·3

* Synonym for roseola infantum.

### Estimation of the mean patient age

The mean patient age of each infectious disease, including KD, varies by season [19]. We pooled the data reported between 2000 and 2010 from both the NSKD and the NESID to circumvent any interference from this seasonal variation. We estimated the crude mean patient age for KD and the seven other paediatric infectious diseases using the following equation [17]: 1
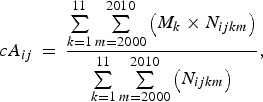
 where *cA*_*ij*_ denotes the crude mean age for the *i*th disease in the *j*th prefecture, *M*_*k*_ represents the midpoint age for the *k*th age group, and *N*_*ijkm*_ represents the number of patients with the *i*th disease from the *k*th age group in the *j*th prefecture in year *m*. The definitions of the 11 age groups (Supplementary Fig. S1) were consistent across the diseases and throughout the study period.

To eliminate interference due to the demographic structure of the data, the mean patient age for each disease was adjusted [17] using the following equation: 2
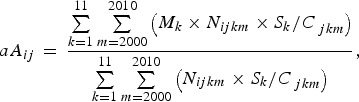
 where *aA*_*ij*_ represents the adjusted mean patient age for the *i*th disease in the *j*th prefecture, *S*_*k*_ represents the proportion of the population in the *k*th age group in the standard population structure (i.e. Japan's population structure in 2000), and *C*_*jkm*_ represents the proportion of the population in the *k*th age group in the *j*th prefecture in year *m*.

In addition, we estimated the national-level mean patient ages using the following equations: 3
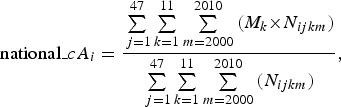

4
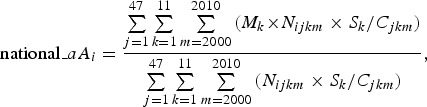
 where national*_cAi* and national*_aAi* represent the crude and adjusted mean patient ages of the *i*th disease at the national level, respectively.

### Estimation of the FOI

The distribution of the FOI often identifies the risk factors for transmission [20] and may also reveal the mode of transmission. In the present study, a surrogate measure of the FOI was estimated as the inverse of the mean patient age as shown in the following equation: 5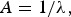 where *A* represents the mean patient age and *λ* represents the FOI for an infectious agent that manifests as an acute illness and confers lifelong immunity [21]. The FOI estimated by equation (5) has been shown to be a good approximation of more elaborate mathematical techniques used to compute the FOI [22]. The FOI and the mean patient age have been shown to be negatively correlated even in a disease caused by multiple viral strains [23].

### Geographical analysis

**Fig. 1.**
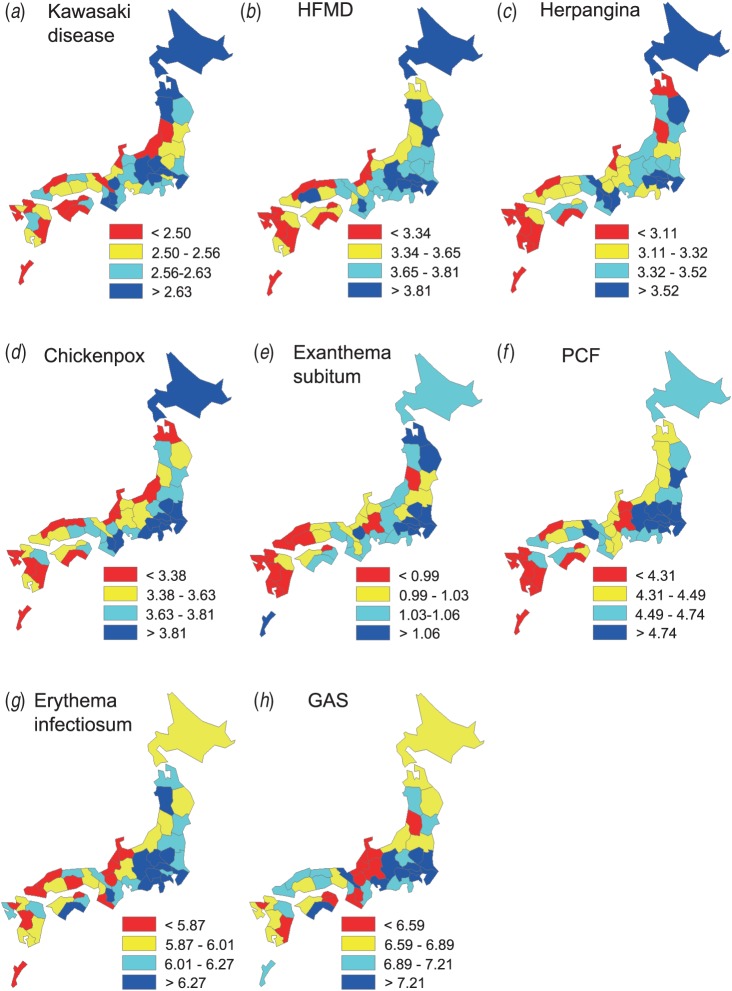
Similarity in the geographical distribution of the crude mean patient age for Kawasaki disease and seven paediatric infectious diseases. The prefectures are categorized from red to blue in ascending order of the crude mean patient age. The distributions of the adjusted mean patient ages were similar to the results presented here. Only data from the main part of each prefecture is presented; minor islands were omitted in this and subsequent figures. Okinawa, which is 650 km away from the main island, is shown close to the main island in this representation. HFMD, Hand, foot and mouth disease; PCF, pharyngoconjunctival fever; GAS, group A streptococcus.

The geographical distribution of the mean patient age was similar between KD and the other paediatric infectious diseases (Fig. 1). To identify the determinants of this shared geographical distribution, we estimated the correlation between the adjusted mean patient ages and climatic/socioeconomic variables. The following climate data was obtained from the University Corporation of Atmospheric Research [24] as described previously [17]: mean temperature (°C), precipitation (mm/day), average vapour pressure (mmHg), and the average vapour pressure deficit (which represents aridity, mmHg). Thirty-seven socioeconomic variables were obtained from the Ministry of Internal Affairs and Communications of Japan [25], including seven demographic variables, eight education variables, eight health variables, seven infrastructure variables, four variables related to the standard of living, and three landscape variables (Supplementary Table S1). The values of these variables, which were surveyed at least three times between 2000 and 2010, were averaged for each prefecture. Mitsubishi Tanabe Pharma (Osaka, Japan), the sole retailer of varicella vaccine in Japan, provided the uptake rate of the varicella vaccine at the prefecture level for each year between 2000 and 2010.

A rank correlation analysis was used to screen the climatic/socioeconomic variables that exhibited a statistically significant (*P* < 0·05) rank correlation with the adjusted mean patient age of all the diseases. We applied multivariate regression analyses to these preselected variables. The adjusted mean patient age was regressed against the explanatory variables using a conventional multivariate linear regression analysis. Then, to account for spatial autocorrelation, we applied a spatial multivariate regression analysis [26] using the following equation: 6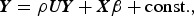 where ***Y*** represents the dependent variable vector, ***X*** represents the independent variable matrix, *ρ* represents the spatial autoregressive parameter, ***β*** denotes the regression coefficient vector, and ***U*** represents the spatial adjacency matrix. Each element of ***U*** was assigned a value of 1 if the two prefectures were geographically adjacent or connected by a bridge or tunnel, and 0 if the two prefectures were not adjacent or connected. The use of the crude mean patient age (*cAij*) instead of the adjusted mean patient age (*aAij*) did not result in qualitatively different results (data not shown). We used Stata v. 11.1 (StataCorp., USA) for all statistical analyses.

### Estimation of the periodicity

We also compared the periodicity between KD and other paediatric infectious diseases. The super-annual periodicity (*T*) in the number of cases was determined by the following equation: 7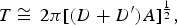 where *D* and *D’* represent the incubation and infectious periods, respectively, and *A* represents the mean patient age of the infectious disease [21]. This relationship holds for persistent infections (e.g. *Plasmodium falciparum*) as well as diseases caused by complex interactions between multiple strains (e.g. dengue haemorrhagic fever) [27]. We tested whether this equation applies to paediatric infectious diseases, and then applied this equation to predict the nature of the aetiological agent for KD.

### Time-series analysis

The monthly number of cases of KD in Japan were available from the NSKD. We converted the weekly number of the seven paediatric infectious disease cases, which were reported from the sentinel paediatric clinics to the NESID between 2000 and 2010, into the monthly number of cases. The monthly number of KD cases and the seven other paediatric diseases were transformed as the proportion to the maximum number of cases recorded during the study period, and were subsequently analysed by the wavelet method [28] using the Morlet wavelet [29]; we followed the standard wavelet procedure used in many recent epidemiological studies (e.g. [23]). We used Mathematica 9.0 (Wolfram, USA) for this analysis.

### Estimation of the probability of KD manifestation

We expressed the proportion of individuals who are naive to the aetiological agent for KD as *V*. The rate of change of *V* at the age of *t* years can be expressed as: 8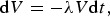 where *λ* represents the FOI. Equation (5) holds for a persistent infection if the incubation period is not very long. Therefore, we solved equation (8) using equation (5) and a condition of *V*(0) = 1 into the following equation: 9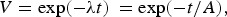 where *A* represents the mean patient age. Based on this equation and epidemiological and demographic data from Japan, we estimated the probability of developing KD when infected with its aetiological agent.

## RESULTS

### Geographical analysis

The distribution of the mean patient age was similar between KD and the other paediatric infectious diseases (Fig. 1); there was a statistically significant rank correlation between all pairs of these diseases with respect to the prefectural mean patient age (Supplementary Table S2). The rank correlations between the adjusted mean patient ages and the climatic/socioeconomic variables were estimated (Supplementary Table S3). This screening process revealed eight variables that were significantly correlated with the adjusted mean patient ages of all the diseases (Table 2). Four of these variables were related to healthcare, and hence were mutually correlated. We excluded non-unique variables to avoid multi-collinearity effects (see uniqueness in Table 2), and included the varicella vaccine uptake rate as a covariate. The final statistical model for each disease was determined by a step-wise addition and elimination of these variables (Table 3).

**Table 2.** Variables which exhibited statistically significant rank correlations with the adjusted mean patient age of Kawasaki disease and paediatric infectious diseases (n = 47)

Variable (unit)	Uniqueness*	Kawasaki disease	Exanthema subitum	Herpangina	HFMD	Chickenpox	PCF	Erythema infectiosum	GAS
Total fertility rate	0·5141	−0·46	−0·46	−0·67	−0·63	−0·60	−0·47	−0·44	−0·33
(dimensionless)		(*P* = 0·0011)	(*P* = 0·0010)	(*P* < 0·0001)	(*P* < 0·0001)	(*P* < 0·0001)	(*P* = 0·0008)	(*P* = 0·0021)	(*P* = 0·0237)
Pupil per class	0·1920	0·44	0·31	0·33	0·40	0·46	0·39	0·30	0·33
(persons)		(*P* = 0·0021)	(*P* = 0·0365)	(*P* = 0·0224)	(*P* = 0·0049)	(*P* = 0·0013)	(*P* = 0·0074)	(*P* = 0·0440)	(*P* = 0·0236)
Hospital	0·1318	−0·45	−0·50	−0·58	−0·64	−0·56	−0·55	−0·48	−0·42
(/100 000)		(*P* = 0·0015)	(*P* = 0·0004)	(*P* < 0·0001)	(*P* < 0·0001)	(*P* < 0·0001)	(*P* = 0·0001)	(*P* = 0·0007)	(*P* = 0·0032)
Bed	0·1038	−0·42	−0·56	−0·61	−0·68	−0·64	−0·64	−0·53	−0·44
(/100 000)		(*P* = 0·0036)	(*P* < 0·0001)	(*P* < 0·0001)	(*P* < 0·0001)	(*P* < 0·0001)	(*P* < 0·0001)	(*P* = 0·0001)	(*P* = 0·0022)
Nurse	0·0173	−0·49	−0·64	−0·72	−0·75	−0·72	−0·69	−0·57	−0·49
(/100 000)		(*P* = 0·0004)	(*P* < 0·0001)	(*P* < 0·0001)	(*P* < 0·0001)	(*P* < 0·0001)	(*P* < 0·0001)	(*P* < 0·0001)	(*P* = 0·0004)
Health insurance	0·2559	−0·40	−0·61	−0·46	−0·52	−0·49	−0·53	−0·61	−0·52
(1 000 000 yen)		(*P* = 0·0050)	(*P* < 0·0001)	(*P* = 0·0013)	(*P* = 0·0002)	(*P* = 0·0005)	(*P* = 0·0001)	(*P* < 0·0001)	(*P* = 0·0002)
Elder ratio	0·1377	−0·3928	−0·4804	−0·4441	−0·4768	−0·5824	−0·4456	−0·3794	−0·4291
(%)		(*P* = 0·0063)	(*P* = 0·0006)	(*P* = 0·0018)	(*P* = 0·0007)	(*P* < 0·0001)	(*P* = 0·0017)	(*P* = 0·0085)	(*P* = 0·0026)
Waste process	0·3582	−0·39	−0·42	−0·43	−0·49	−0·54	−0·45	−0·36	−0·37
(%)		(*P* = 0·0073)	(*P* = 0·0030)	(*P* = 0·0021)	(*P* = 0·0005)	(*P* = 0·0001)	(*P* = 0·0017)	(*P* = 0·0139)	(*P* = 0·0100)

HFMD, Hand, foot and mouth disease; PCF, pharyngoconjunctival fever; GAS, group A streptococcus.

* Uniqueness was estimated by factor analysis. A variable of smaller ‘uniqueness’ can be largely explained by a linear combination of the other variables.

**Table 3.** Conventional and spatial regression models to explain adjusted mean patient ages of Kawasaki disease and paediatric infectious diseases

Variable*	Kawasaki disease	Exanthema subitum	Herpangina	HFMD	Chickenpox	PCF	Erythema infectiosum	GAS
**Conventional regression**								
Total fertility rate	−0·29	−0·13	−1·9	−1·7	−0·92	−1·2	−0·69	n.s.
	(*P* < 0·0001)	(*P* = 0·0207)	(*P* < 0·0001)	(*P* < 0·0001)	(*P* = 0·0053)	(*P* = 0·0019)	(*P* = 0·0159)	
Health insurance	n.s.	−1·3	−3·1	−4·2	−2·2	−8·0	−6·2	−11
		(*P* < 0·0001)	(*P* = 0·0228)	(*P* = 0·0020)	(*P* = 0·0151)	(*P* < 0·0001)	(*P* < 0·0001)	(*P* = 0·0001)
Varicella	n.s.	n.s.	n.s.	n.s.	1·1	n.s.	n.s.	n.s.
Vaccination					(*P* = 0·0077)		
*R* ^2^	0·34	0·45	0·56	0·58	0·62	0·46	0·44	0·29
**Spatial regression**								
Total fertility rate	−0·26	−0·16	−0·18	−0·16	−0·66	−1·0	−0·62	n.s.
	(*P* < 0·0001)	(*P* = 0·0022)	(*P* < 0·0001)	(*P* < 0·0001)	(*P* = 0·0281)	(*P* = 0·0030)	(*P* = 0·0195)
Health insurance	n.s.	−0·15	n.s.	−2·7	n.s.	−6·3	−5·7	−11
		(*P* < 0·0001)		(*P* = 0·0253)		(*P* = 0·0002)	(*P* < 0·0001)	(*P* = 0·0001)
Varicella	n.s.	n.s.	n.s.	n.s.	1·4	n.s.	n.s.	n.s.
Vaccination					(*P* < 0·0001)		
*R* ^2^	0·41	0·50	0·58	0·66	0·62	0·53	0·45	0·29

HFMD, Hand, foot and mouth disease; PCF, pharyngoconjunctival fever; GAS, group A streptococcus; n.s., not significant.

* Although five variables (total fertility rate, pupils per class, health insurance, waste process, and chickenpox vaccination) were incorporated into the multivariate analysis, only total fertility rate, health insurance, and chickenpox vaccination remained as statistically significant contributor(s).

**Fig. 2.**
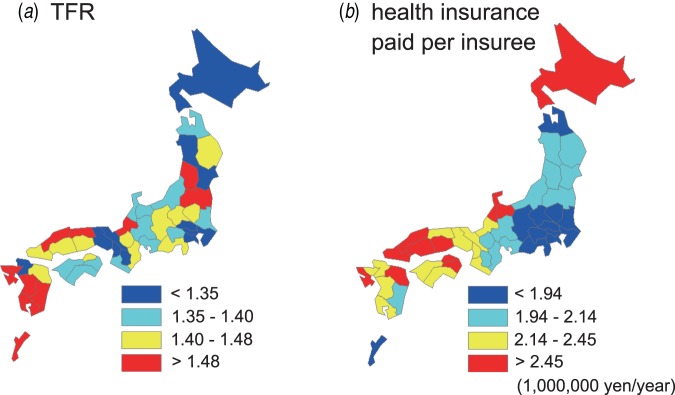
Distribution of socioeconomic factors that were correlated with the mean patient age for each prefecture. The prefectures were categorized from red to blue in descending order for (*a*) the total fertility rate (TFR) and (*b*) the health insurance paid per insuree.

The health insurance paid per insuree remained as a significant negative contributor to the adjusted mean patient age for most of the diseases. The total fertility rate (TFR) remained as a significant negative contributor to the adjusted mean patient age for all the diseases except group A streptococcus (GAS). These results suggest that the availability/utilization of health services, and more importantly, the TFR (Fig. 2) are critical factors in the transmission of KD and paediatric infectious diseases. Notably, Okinawa prefecture, which has the largest TFR and lowest health insurance paid per insuree, emerged as an outlier for some diseases (Supplementary Figs S2, S3).

### Seasonality

**Fig. 3.**
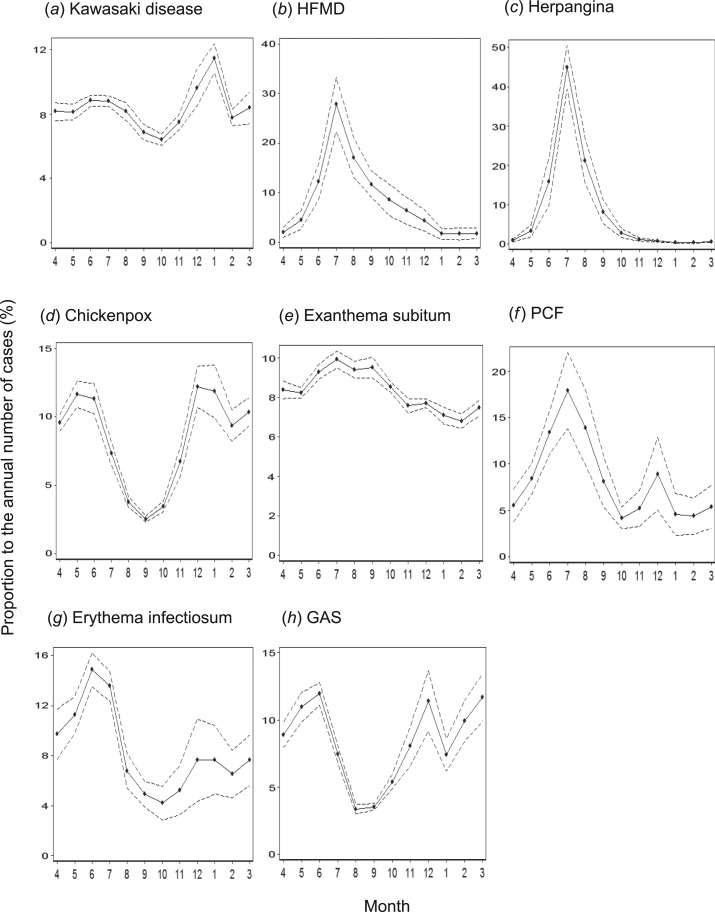
Seasonality of Kawasaki disease and other paediatric infectious diseases. The proportion of the number of cases in a month to the annual number of cases was estimated for each year between 2000 and 2010 and averaged across these 11 years. The dashed lines indicate the standard deviation. The x-axis starts in April to reflect the beginning of the Japanese academic school year. HFMD, Hand, foot and mouth disease; PCF, pharyngoconjunctival fever; GAS, group A streptococcus.

We compared the seasonality across diseases. We presented our data starting in April to reflect the beginning of the Japanese academic school year (Fig. 3). Hand, foot, and mouth disease (HFMD) and herpangina exhibited a unimodal distribution with a sharp peak during the summer (July). GAS exhibited trimodal seasonality with three major peaks in June, December, and March. KD, chickenpox, pharyngoconjunctival fever (PCF), and erythema infectiosum exhibited a bimodal distribution with two major peaks, one in June and the other in December/January, as well as a subtle minor peak in March.

### Super-annual periodicity

**Fig. 4.**
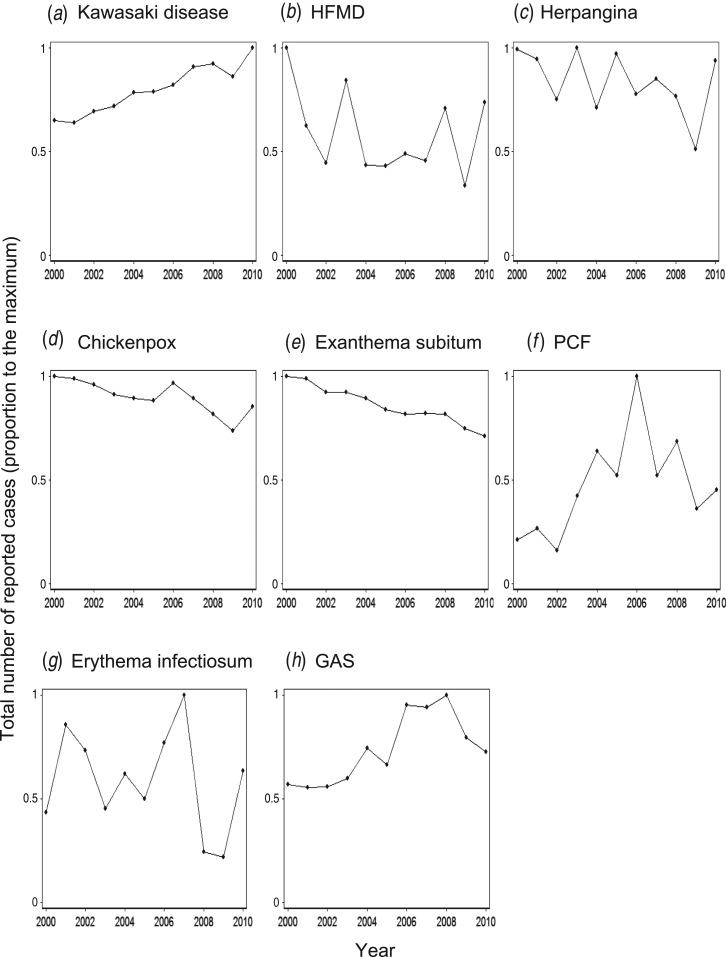
Annual time series of Kawasaki disease (KD) and other paediatric infectious diseases in Japan between 2000 and 2010. The annual number of cases of KD and other paediatric infectious diseases was expressed as a proportion to the maximum number of reported cases. PCF, pharyngoconjunctival fever; GAS, group A streptococcus.

**Fig. 5.**
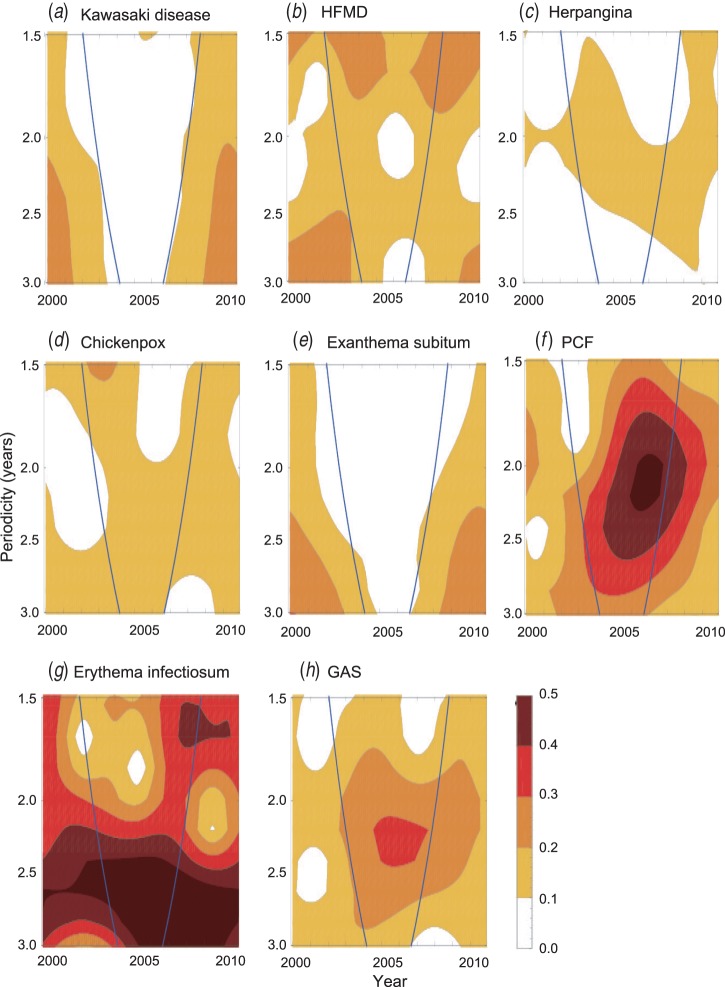
Super-annual periodicities of KD and other paediatric infectious diseases as detected by the wavelet analysis. The blue lines indicate the 95% confidence limit of the wavelet analysis. The area outside the blue lines is unreliable due to the edge effect. PCF, pharyngoconjunctival fever; GAS, group A streptococcus.

**Fig. 6.**
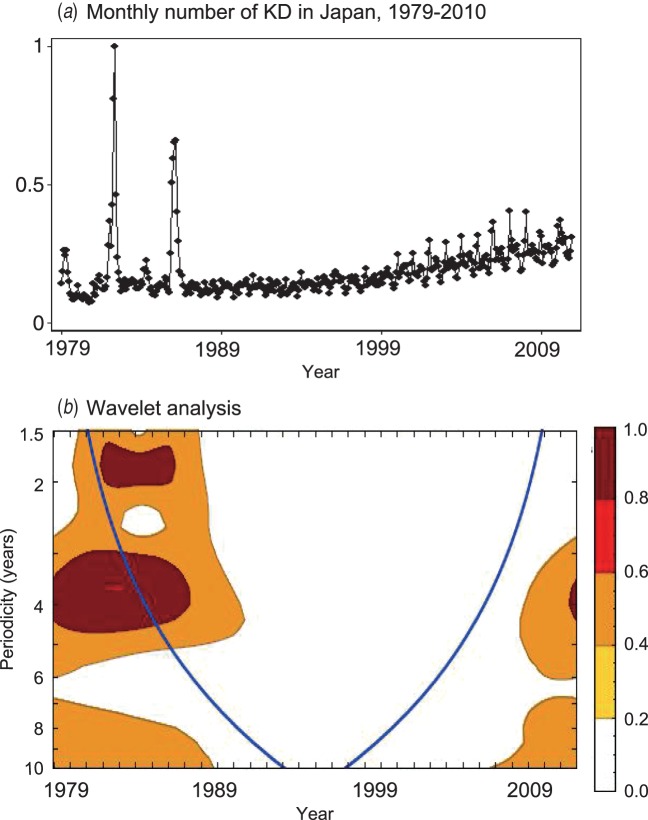
Monthly number of Kawasaki disease (KD) cases between 1979 and 2010. The monthly number of KD cases recorded between 1979 and 2010 in Japan are expressed as the proportion to the maximum number of cases (*a*). The results of the wavelet analysis applied to this time series are plotted in (*b*).

All the diseases examined in the present study, except KD and exanthema subitum, exhibited substantial fluctuations in the number of cases from year to year (Fig. 4). The wavelet analysis revealed that all the diseases except KD and exanthema subitum exhibited some coloured area, which implies that super-annual periodicity was detected in the corresponding period (Fig. 5). We obtained the incubation period (*D*) and infectious period (*D’*) of each disease from Richardson *et al.* [30] (Table 4) and used the adjusted mean patient age (*A*) (Table 1) to test whether the super-annual periodicity could be explained by equation (7). Most of the super-annual periodicities estimated by the wavelet analysis were consistent with the predicted values (Table 4). We could not judge whether the prediction for GAS was successful because *D* + *D’* was ambiguous for this disease. We also predicted *D* + *D’* for KD using equation (7). Because *T* was longer than 10 years after 1987 (Fig. 6) and *A* is 2·4 years, the *D* + *D’* for KD was estimated to be larger than 1·1 years. Since *D* for KD is approximately 10 days [31], *D’* for KD therefore is likely to be longer than 1 year. We will report the hypothesis elsewhere that the epidemics in 1979, 1982, and 1986 were driven by dynamics unique to these years (Y. Nagao, unpublished data ).

**Table 4.** The lengths of predicted and actual super-annual periodicities (T)

	Incubation period (*D*)	I Infectious period (*D*’)	*D* + *D*’	*T* predicted by equation (2)	*T* measured in actual time series by wavelet analysis
Exanthema subitum	10–15 d	Lifelong	>10 yr	>20 yr	>3·0 yr
Herpangina	3–5 d	<7 d	11 d	2·0 yr	2·2 yr
HFMD	3–5 d	<7 d	11 d	2·1 yr	1·7 yr
Chickenpox	11–20 d	−1 to +2 d	16 d	2·5 yr	>2·0 yr
PCF	3–29 d	<2 wk	16 d	2·8 yr	2·2 yr
Erythema infectiosum	13–18 d	−6 d to –3 d	9 d	2·4 yr	2·6 yr
GAS	12 h–5 d	(1) untreated: 1–12 mo. (2) treated: <4 d	(1) 3 mo. (2) 8 d	(1) 8·3 yr (2) 2·5 yr	2·3 yr

HFMD, Hand, foot and mouth disease; PCF, pharyngoconjunctival fever; GAS, group A streptococcus; d, days; wk, weeks; mo., months; yr, years.

### Probability of KD manifestation

Between 2006 and 2010, the average annual number of KD cases in Japan was 7699 for children aged between 0 and 2 years and 10 115 for children aged between 0 and 4 years. During the same period, the average number of live births was 1 082 997 per year and the death rate for children aged <5 years was 3667 per year. From these numbers, we estimated that approximately 0·7% and 0·9% of Japanese children had been diagnosed with KD by their third and fifth birthdays, respectively. By substituting *A* in equation (9) with the mean age of KD (i.e. 2·4 years), the proportion of individuals who experienced infection(s) with the aetiological agent for KD (1 *– V*) was computed as 71% and 88% at *t* = 3 and *t* = 5 years, respectively. Therefore, only about 1% of infections with the KD agent(s) manifested with symptoms of KD.

## DISCUSSION

The geographical distribution of the FOI of KD and other paediatric infectious diseases was similar across Japan. We identified the availability/utilization of health services and the TFR as the main determinants of this shared geographical structure. Particularly, the TFR remained as a statistically significant risk factor to the FOI of KD and all the viral diseases, after accounting for the possible confounders and the spatial autocorrelation.

Indeed, there have been numerous reports that health services facilitate the transmission of paediatric infectious diseases [32, 33]. In addition, the causal relationship between the TFR and the FOI is intuitive: a higher TFR implies a larger number of siblings within a household, which increases the probability of sibling-to-sibling infection. Previous studies have also identified the importance of sibling-to-sibling infections for KD [31, 34].

The finding that sibling-to-sibling infection and, to a lesser extent, hospital infection emerged as important routes of transmission for KD and other paediatric infectious diseases in this study suggests that KD shares a mode of transmission with other paediatric infectious diseases, i.e. close contact with an infected individual. KD also exhibited a bimodal seasonality similar to other viral infections such as chickenpox and erythema infectiosum, each of which is caused by a single agent. Therefore, we cannot assert that KD is caused by multiple agents based solely on its multimodal seasonality. The seasonality of a paediatric infectious disease is largely determined by climate and the school term [35, 36]. Indeed, the troughs in the seasonality of GAS occurred in April, September, and January, immediately following school/kindergarten holidays in the spring (late March to early April), the summer (late July to late August), and the winter (late December to early January), respectively. This finding suggests that these troughs were due to the separation of children during the holidays. This finding also suggests that the bimodal seasonalities of KD, chickenpox, PCF, and erythema infectiosum were affected by the school term. Our finding that KD shares a similar seasonality pattern to other paediatric diseases suggests that the transmission routes for these diseases are common. Taken together, these results suggest that KD is not likely to be transmitted through a novel route such as a transcontinental wind [37, 38].

One notable outlier in our regression analysis was Okinawa prefecture, a tropical prefecture composed of 160 islands located 650 km from Japan's mainland. Host genetic heterogeneity is an unlikely explanation for this observation, because the genetic structure of the Okinawan population is similar to other populations within Japan [39]. The tropical climate of Okinawa may contribute to this observation [40]. Alternatively, due to the remoteness and multi-island structure of Okinawa, the transient extinction of a pathogen may be more frequent than on the mainland of Japan [41].

One major limitation of the present study was that the data about the paediatric infectious diseases was derived from sentinel paediatric clinics. The allocation of sentinel clinics is not precisely proportional to the population size. However, no factor was suggested as a possible source of bias in estimating the mean patient age from the sentinel data. Therefore, we believe that our analyses were not affected significantly by the use of sentinel data.

Another possible refutation against the present analysis is that our statistical analysis may have been flawed by ecological confounding. However, we believe that the identified contributions of fertility rate and hospital utilization to the FOI are not statistical artefacts. Four of the eight variables, which were preselected from a large number of variables during the initial screening process, were related to the utilization of health services (Table 2). This result strongly suggests that health services play an important role in determining the FOI. In addition, the varicella vaccine uptake rate remained as a statistically significant predictor of risk reduction on the FOI of chickenpox only (Table 3). This result validates our analytical procedure, because vaccination is assumed to decrease the FOI [21, 42]. Moreover, the fertility rate (or birth rate) is one of the most important factors that determine the epidemiology of paediatric infectious diseases [28].

Our mathematical assumptions may seem to contain excessive approximations. For example, equation (5) presumes a fixed longevity (or Type I survival). This, however, is a well-founded approximation. First, Type I survival is more suitable ‘for humans, especially in developed countries’ than Type II survival, which assumes age-independent mortality [21]. Second, regardless of whether Type I or II survival is assumed, the mean patient age can effectively approximate the inverse of the FOI, if the mortality is very small as in Japan. Equation (8), which has been used in epidemiological studies [43], presumes continuous susceptibility to a given disease from birth and complete endemicity of the disease agent. Therefore, our prediction based upon equation (8) should be tested in future serological surveys once the aetiological agent for KD is identified.

Given that any infectious disease has finite values for the incubation and infectious periods (i.e. *D* + *D*’), equation (7) predicts that any infectious disease will have a finite super-annual periodicity (*T*). However, among the diseases examined in this study, only KD and exanthema subitum exhibited the least evidence for super-annual periodicities. This finding suggests that the super-annual periodicities of these diseases are very long, and hence the sum of the incubation period and infectious period of these diseases is large [equation (7)]. Consistent with this finding, the viral agent for exanthema subitum persists across the lifespan [44]. Therefore, it would not be surprising if the aetiological agent for KD also persists for a long period. Our finding is consistent with a previous report that the agent (or at least one of the agents) for KD is capable of persistent infection [19].

The low mean patient age, and therefore the strong FOI of KD, led to an estimation that only about 1% of infections with the KD agent(s) manifest with symptoms of KD. Our finding that KD may persist over a long period and cause frequent asymptomatic infections predicts that the aetiological agent(s) for KD exists in KD patients as well as in many healthy individuals. Therefore, an epidemiological study to determine whether a particular microbe is a causative agent for KD will require a large sample size to detect subtle difference in the prevalence of the microbe between cases and controls. In addition, the possibility that sibling-to-sibling infection is an important route of transmission for KD implies that the number of siblings and the rank of the patient among them should be considered as covariates in epidemiological analyses.

## Supplementary material

For supplementary material accompanying this paper visit http://dx.doi.org/10.1017/S0950268815001223. click here to view supplementary material
