# Supplementary material for: Predicting the characteristics of the aetiological agent for Kawasaki disease from other paediatric infectious diseases in Japan
Source: Epidemiol Infect. 2015 Jul 23;144(3):478–92. doi: 10.1017/S0950268815001223 (PMC4714300; doi:10.1017/S0950268815001223)
Supplement: Supplementary file 1 [file S0950268815001223sup001.zip › SUPMAT Figure and Table legends.docx]

**Figures and Tables for the Supplementary Material**

**Fig. S1.** Age distribution of KD and other paediatric infectious diseases. Each distribution represents the proportion of cases in each age group relative to the total number of cases.

**Fig. S2.** Negative correlations between the total fertility rate and the mean patient age for KD and other paediatric infectious diseases. Each dot corresponds to one of the 47 prefectures. The prefecture with the largest total fertility rate, Okinawa, was an outlier in some of these negative correlations.

**Fig. S3.** Negative correlations between the health insurance paid per insuree and the mean patient age for KD and other paediatric infectious diseases. Each dot corresponds to one of the 47 prefectures. The prefecture with the smallest health expenditure, Okinawa, was an outlier in some of these negative correlations.

Table S1. *Definitions of the climatic, demographic, and socioeconomic variables examined in the current study.*

Table S2. *The rank correlation between the adjusted mean patient ages of KD and paediatric infectious diseases at the prefectural level (n=47).*

Table S3. *The rank correlations between the climatic/socioeconomic variables and the adjusted mean patient age.*
