# Supplementary material for: Predicting the characteristics of the aetiological agent for Kawasaki disease from other paediatric infectious diseases in Japan
Source: Epidemiol Infect. 2015 Jul 23;144(3):478–92. doi: 10.1017/S0950268815001223 (PMC4714300; doi:10.1017/S0950268815001223)
Supplement: Supplementary file 1 [file S0950268815001223sup001.zip › epi_infect_Geography_pediatric_diseases_d_Sup_Table_S2_disease_vs_disease.doc]

**Epidemiology and Infection**

**Predicting characteristics of the aetiologic agent for Kawasaki disease from other paediatric infectious diseases in Japan**

**Y. Nagao, C. Urabe, H. Nakamura, N. Hatano**

**Supplementary Material**

**Supplementary Table S2. Rank correlation between adjusted mean patient ages of KD and pediatric infectious diseases, at the prefectural level (n=47).**

|  | KD | Exanthema subitum | Herpangina | HFMD | Chickenpox | PCF | Erythema infectiosum |
| --- | --- | --- | --- | --- | --- | --- | --- |
| Exanthema subitum | 0.51  (P=0.0003) |  |  |  |  |  |  |
| Herpangina | 0.59  (P<0.0001) | 0.58  (P<0.0001) |  |  |  |  |  |
| HFMD | 0.61  (P<0.0001) | 0.54  (P=0.0001) | 0.85  (P<0.0001) |  |  |  |  |
| Chickenpox | 0.55  (P=0.0001) | 0.55  (P=0.0001) | 0.80  (P<0.0001) | 0.87  (P<0.0001) |  |  |  |
| PCF | 0.49  (P=0.0004) | 0.59  (P<0.0001) | 0.81  (P<0.0001) | 0.83  (P<0.0001) | 0.80  (P<0.0001) |  |  |
| Erythema infectiosum | 0.45  (P=0.0017) | 0.52  (P=0.0002) | 0.54  (P=0.0001) | 0.65  (P<0.0001) | 0.63  (P<0.0001) | 0.56  (P<0.0001) |  |
| GAS | 0.30  (P=0.0375) | 0.58  (P<0.0001) | 0.49  (P=0.0005) | 0.50  (P=0.0003) | 0.49  (P=0.0005) | 0.64  (P<0.0001) | 0.65  (P<0.0001) |
